# The political economy of adolescent mental health in Kenya

**DOI:** 10.1093/heapol/czaf057

**Published:** 2025-08-29

**Authors:** Albert Tele, Darius Nyamai, Yusra Ribhi Shawar, Vincent Nyongesa, Samuel Kiogora, Stefan Swartling Peterson, Georgina Obonyo, Pim Cuijpers, Manasi Kumar

**Affiliations:** Department of Clinical, Neuro and Developmental Psychology, Vrije Universiteit Amsterdam, Van der Boechorststraat 7, 1081 BT Amsterdam, The Netherlands; Department of Health Wellness and Nutrition, City Hall Annex, 3rd Floor, Room 305, Nairobi City County, Kenya; Paul H. Nitze School of Advanced International Studies, Bloomberg School of Public Health, Johns Hopkins University, 615 N. Wolfe St. Baltimore, MD 21205, United States; Department of Psychiatry, University of Nairobi, Kenyatta National Hospital (KNH), P.O. Box 19676 – 00202, Nairobi, Kenya; Tabasamu Café Nairobi, Muhoho Avenue, South C, Nairobi, Kenya; Department of Public Health Medicine, School of Nursing & Public Health, College of Health Sciences, University of Kwa-Zulu-Natal, Howard College Campus, George Campbell Building West Wing, Private Bag X54001, Durban 4000, South Africa; Department of Global Public Health, Karolinska Institutet, SE-171 77 Stockholm, Sweden; School of Public Health; College of Health Sciences, Makerere University, New Mulago Gate Rd, P.O. Box 22864, Kampala, Uganda; Tabasamu Café Nairobi, Muhoho Avenue, South C, Nairobi, Kenya; Department of Clinical, Neuro and Developmental Psychology, Vrije Universiteit Amsterdam, Van der Boechorststraat 7, 1081 BT Amsterdam, The Netherlands; Department of Population Health, NYU Grossman School of Medicine, 180 Madison Avenue, NY 10016, United States

**Keywords:** adolescent mental health, Kenya, policy development, stakeholder perceptions, barriers, stigma, advocacy

## Abstract

Adolescent mental health remains a critical yet under-prioritized issue in low- and middle-income countries (LMICs) like Kenya, where resource limitations, stigma, and systemic barriers hinder access to care. While policies and strategies such as Kenya's Mental Health Action Plan (2021–2025) exist on paper, their implementation is constrained by limited resources and a weak mental health service delivery infrastructure. This qualitative descriptive study examines the perspectives of mental health actors and youth advocates on the development and implementation of adolescent mental health policy in Kenya. Using a political economy analysis, we conducted 15 key informant interviews (KIIs) and analyzed observational field notes from a Google Jam board exercise to explore factors that enable or impede the prioritization of adolescent mental health policy and care. Thematic analysis was guided by Shiffman and Smith's policy framework, focusing on four domains: actor power, ideas, political context, and issue characteristics. Findings reveal significant barriers, including the exclusion of adolescents from decision-making, limited family involvement, weak policy formulation, and the destabilizing effects of government transitions. Stigma, poverty, and chronic underfunding further hinder progress, despite ongoing strategic efforts. Comparisons with other LMICs indicate that these challenges are widespread, underscoring the need for localized, inclusive, and well-coordinated approaches. Addressing these issues will require strong political commitment, increased youth-led advocacy, and sustained investment in mental health services. By prioritizing adolescent mental health, Kenya can move toward a more equitable and effective mental health system that supports the wellbeing of its youth.

Key messages

- Adolescent mental health remains under-prioritized in Kenya despite the existence of policy frameworks like the Mental Health Action Plan (2021–2025). Underfunding, stigma, and weak infrastructure continue to hinder effective care.
- Structural and systemic barriers limit policy implementation, including the exclusion of adolescents from decision-making, inadequate family involvement, and disruptions due to government transitions.
- Socioeconomic factors and stigma further restrict access to care, with poverty, cultural beliefs, and limited awareness acting as key deterrents to help-seeking among adolescents.
- Strengthening political commitment, youth-led advocacy, and sustained investment is essential to overcoming these challenges and ensuring adolescent mental health is a national priority.

## Introduction

Mental health represents a significant global burden, with adolescents in low- and middle-income countries (LMICs) disproportionately affected (Patton *et al.* 2016).

Mental disorders are among the leading causes of disability worldwide, and adolescence—a critical period developmental stage typically ranging from ages 10 to 19, marked by rapid physical growth, emotional fluctuations, identity formation, and increasing social pressures—is particularly vulnerable to the impacts of conditions like depression, anxiety, and behavioral disorders (Vigo *et al.* 2016, WHO 2024). Adolescents account for 23% of the population in sub-Saharan Africa, where mental health challenges are compounded by limited resources, stigma, and socioeconomic barriers (Blum *et al.* 2012, Vigo *et al.* 2016, Iemmi 2022, WHO 2024). Globally, 10%–20% of adolescents experience mental health issues; yet, access to care remains alarmingly inadequate (McGorry *et al.* 2022, UNICEF 2022).

In recent years, there has been growing recognition of adolescent mental health as a priority in LMICs, with countries like Kenya developing policies such as the Mental Health Action Plan (2021–2025) (Ministry of Health 2021). This plan and other national advocacy and policy initiatives aim to address mental health challenges through improved service delivery and system strengthening.

However, systemic issues—including underfunding, weak infrastructure, and pervasive stigma—continue to hinder effective implementation and equitable access to care (Collishaw 2015, Benton *et al*. 2021).

In addition, there is insufficient political will, national prioritization, and effective policy and service implementation for the issue (Kumar *et al.* 2021, Memiah *et al.* 2022, Chemonges 2024). In Kenya, socioeconomic challenges such as poverty and violence exacerbate the adolescent mental health crisis, and national policies often fail to address these specific needs (Patel *et al.* 2018, Kenya Adolescent Mental Health Group 2024). The Kenyan Task Force on Mental Health reported that 25% of outpatients and 40% of inpatients suffer from mental illnesses; yet, 75% of Kenyans, especially adolescents, lack access to adequate care (Ministry of Health 2020). Limited resources allocated to adolescent mental health programs, combined with a lack of political will, have impeded the effective implementation of services (WHO 2021).

While existing literature highlights the burden of adolescent mental health issues in LMICs, there is limited research examining the political and systemic factors that influence policy prioritization, development, and implementation—particularly in the Kenyan context. Furthermore, few studies incorporate the perspectives of diverse stakeholders, including youth advocates, service providers, and individuals with lived experience.

Accordingly, this study seeks to map the views of a diverse group of stakeholders, including national and county-level mental health policymakers, service providers (e.g. psychiatrists, psychologists), youth mental health advocates, and individuals with lived experience on the prioritization, development, and implementation of policies and services for adolescent mental health in Kenya.

This study examines the political economy of adolescent mental health in Kenya by exploring how various stakeholders perceive, engage with, and influence the formulation, prioritization, and implementation of adolescent mental health policies. It seeks to understand how their actions, relationships, and institutional contexts shape the policy landscape and contribute to current responses to adolescent mental health needs in the Country.

The specific objectives are the following:

1. To explore stakeholders’ perceptions of the current adolescent mental health policy formulation and implementation in Kenya.
2. To explore stakeholders’ engagement and influence in the prioritization of the adolescent mental health agenda in Kenya.

The study is guided by the following research question: How do diverse stakeholders engage with and influence the prioritization, development, and implementation of adolescent mental health policies and services in Kenya?

## Materials and methods

This study was conducted in Kenya at both the national and County levels. At the county level, we purposefully selected three counties—Meru, Tharaka-Nithi, and Nairobi—to reflect diversity in geographic location, urban–rural distribution, and health system capacity. These counties were chosen based on their involvement in youth-focused programming, existing mental health initiatives, and accessibility to key informants across different levels of the health and policy system. Under Kenya's devolved system of Government, introduced in 2010, responsibility for delivering health services was transferred from the national to the 47 county governments. County governments are now responsible for planning, financing, and managing most health services, including mental health. The national Government retains a policy and regulatory role, while counties implement service delivery. This governance structure means that county-level leadership and priorities significantly influence how national policies—such as those related to adolescent mental health—are interpreted and implemented on the ground.

Data for this qualitative descriptive study were collected between October and December 2022. Data sources included 15 key informant interviews (KIIs) with stakeholders across different sectors, as well as observational field notes from a participatory online session conducted using Google Jamboard.

Participants were selected using purposeful sampling and snowballing through a network of referrals. The participants were stratified by sector (public, private, third sectors, and multisector partnerships) to capture population heterogeneity. They were mental health policy makers at both National and devolved County government levels who develop strategies and action plans. We also interviewed mental health care workers who practice as psychiatrists and psychologists because they are stakeholders in the implementation of mental health policy and guidelines. Mental health advocates/champions were also interviewed as consumers of the services. These were mainly service users with lived experience who are currently engaging in creating mental health awareness in the community while advocating for equity in the accessibility of quality services. These participants were identified through the researchers’ professional networks, consultation of relevant national meetings and organizational reports, and review of Government, donor, and NGO documents, as well as published documents. Snowball sampling was used to recruit additional participants who participated in adolescent mental health policy development and implementation by contributing to policy documents in stakeholder meetings that advocate for adolescent mental health issues. To ensure the inclusion of youth voices, we engaged youth advocates who have advocated for adolescent health and/or played a role in adolescent health policy. Youth advocates aged 15 years and above were eligible to participate in this study; however, no participants under the age of 18 were ultimately interviewed. Ethical approval had been obtained to include minors, with provisions for both parental/guardian consent and minor assent in accordance with national and institutional research ethics guidelines.

Fifteen participants agreed to be interviewed. Key informant interviews (KIIs) were conducted with three national-level mental health policy specialists, seven youth mental health advocates, three county-level policy and mental health program specialists involved in the coordination and implementation of mental health initiatives, and two mental health specialists and educators (i.e. psychologists or psychiatrists) who also provide training and education (see Table 1). Informed consent was obtained from participants before the interview, either in writing or oral form. Each interview lasted on average 45 minutes and was conducted face-to-face and virtually, via a Zoom link. Interviews were digitally recorded and guided by a semi-structured topic guide comprising open-ended questions, developed by the researcher.

**Table 1.** Participants and their cadres.

Sector	Number
National-level mental health policy specialists	3
Youth mental health advocates	7
County (sub-national level) policy and Mental Health program specialists	3
Mental health specialists and educators	2
**Total**	15

While the broader framing of the study drew from historical global efforts to understand the political prioritization of mental health since 1990, the qualitative interviews specifically focused on current national and County government processes influencing the prioritization of adolescent mental health in Kenya. The perspectives of adolescents (aged 10–19), alongside those of policymakers and stakeholders, were collected to explore lived experiences, perceived policy gaps, and implementation challenges relevant to their mental health.

The open-ended nature of the questions allowed the researcher to probe for deeper insights and clarify responses, facilitating a more nuanced understanding of the issues under investigation. The data were audio-recorded and transcribed verbatim in English. Observational field notes were drawn from a participant observation conducted during a virtual participatory session held from October to December 2022 using Google Jamboard, where youth and stakeholder participants discussed priority issues in adolescent mental health. Facilitators used a structured observation guide to capture key themes, participant interactions, and areas of consensus or ambiguity. These field notes were subsequently compared with the KII transcripts to help clarify emergent issues, ensure consistency, and enhance interpretation of the interview data. The open-ended interview guide used during data collection is provided as Supplementary Material.

### Data analysis

Data were analyzed using a hybrid approach that combined inductive thematic analysis (Braun and Clarke 2006) with theory-informed interpretation guided by the Shiffman and Smith (2007) policy framework. An initial codebook was developed *a priori* based on the key informant interview (KII) topic guides. This codebook was then refined iteratively through careful review of transcripts, with emergent codes added as new themes arose from the data.

The transcripts were coded using QSR NVivo Version 14, and data were analyzed using a constant comparison method to identify patterns and themes aligned with the study objectives. After the initial inductive coding and theme development, the themes were organized and interpreted through the lens of the Shiffman and Smith (2007) framework, which focuses on understanding the determinants of political priority in global health.

This framework comprises four main categories:

1. **Actor power**—strength and cohesion of policy communities, leadership, guiding institutions, and civil society mobilization. In this study, the actors would refer to policy makers, health care workers, mental health advocate groups, NGOs, religious organization, caregivers, and most importantly the persons with mental illness.
2. **Ideas**—how the issue is internally framed within the policy community and externally communicated to the public and policymakers.
3. **Political contexts**—policy windows and broader political and institutional environments that shape prioritization.
4. **Issue characteristics**—perceived severity of the issue, availability of credible indicators, and the existence of effective interventions.

(See Table 2 for a detailed summary of these categories and associated sub-components.)

**Table 2.** Framing issues using the Shiffman and Smith model.

	Theme	Summary description
Policy level issues		
Ideas	Inadequate policy addressing adolescent mental health.	There is a knowledge gap in addressing specific adolescent mental health needs due to an inadequate dedicated policy framework.
Actor power	Exclusion of adolescents and caregivers voice in mental health policy decisions	Exclusion of adolescents and caregivers’ contribution in decision-making process undermines the legitimacy of the proposed policies.
Political contexts	Government transition and policy changes	Transition of governance interferes with sustainability of created momentum of policy issues
Issue characteristics	Insufficient data to quantify the mental health burden	Insufficient data to quantify and estimate the real burden of adolescent mental health is a challenge in prioritizing adolescent mental health at policy level
	Low level of funding for mental health programs	Funding of mental health is significantly low in the country. There is a need to advocate for more investment at the policy level
Implementation level issues		
Ideas	Positioning issue—Inadequate priming of adolescent mental health agenda	The inadequate framing of the adolescent mental health agenda has made it harder to address the problem coherently at the level of implementation.
	Inadequate mental health knowledge	The existing knowledge gap regarding adolescent mental health, as well as a negative perception is a hindrance for effective programs implementation.
Actor power	Inadequate coordinated efforts for coalition building on implementation of adolescent mental health programs	Limited coordination among the stakeholders poses a significant challenge in advancing the adolescent mental health agenda.
Issue characteristics	Persistent stigma around mental ill-health	Stigma is a hindrance in the implementation of adolescent mental health care
	Inadequate access to effective and affordable mental health services	Inadequate dedicated adolescent mental health infrastructure and the uneven distribution of skilled personnel are major challenges to equitable service access and affordability.

The Shiffman and Smith (2007) framework was used primarily as an interpretive tool during the analysis phase. After an initial round of inductive thematic analysis, the emergent themes were mapped onto the four categories of the framework—actor power, ideas, political contexts, and issue characteristics—to facilitate interpretation and structure the findings. While the framework did not inform the development of the interview topic guide in a strict deductive sense, the topic areas broadly aligned with its core domains. Given this approach, a hybrid method combining thematic analysis with elements of framework analysis was adopted, enabling both inductive insights and theory-informed interpretation.

Verbatim quotes were extracted to illustrate key themes and were anonymized to protect participants’ confidentiality. DN led the initial coding and theme development, and AT independently reviewed and confirmed the final thematic structure.

### Use of AI tools

During the preparation of this manuscript, Grammarly was utilized to assist with language refinement. All AI-generated content was thoroughly reviewed and verified by the authors to ensure accuracy, coherence, and alignment with the study’s objectives and results. No AI tools were employed in the collection, generation, or statistical analysis of the data. The authors take full responsibility for the content of the manuscript, including all sections developed with the assistance of AI.

## Results

The results section is presented in two broad parts: policy-level and implementation-level findings. Within each section, emergent themes are analyzed through the lens of the Shiffman and Smith (2007) framework, with particular attention to the domains of ideas, actor power, political context, and issue characteristics, as evidenced in participants’ narratives.

Policy formulation, in this context, refers to the decision-making processes concerning, which adolescent mental health interventions to prioritize and adopt. Implementation refers to the operationalization of these decisions, including resource allocation, delivery mechanisms, and stakeholder engagement at various levels.

### Policy-level issues

#### Ideas

##### Absence of a specific adolescent mental policy

Participants indicated that there is currently no specific adolescent mental health policy in place. They were explaining the existing gap in addressing specific needs in adolescent mental health. A participant commented, “*To be honest I am not aware of specific policy for adolescent mental health—if it does exist, I do not know about it” (KII with youth mental health advocates-CM).*

Another participant echoed this view, emphasizing that while a national mental health policy exists, it does not explicitly cater to adolescents: “*I think there are no specific mental health policies that target adolescents. I think what we have is the mental health policy” (KII with youth mental health advocates—GO & MW).*

This perception was supported by findings from our review of national and county-level policy documents. Although several health policy frameworks reference mental health—such as the Kenya Mental Health Policy (2015–2030) (Ministry of Health Kenya 2015a), the Adolescent Sexual and Reproductive Health Policy (Ministry of Health Kenya 2015b), and various County Integrated Development Plans—adolescent mental health is either absent or only mentioned briefly without concrete strategies or dedicated resource allocations. In most cases, adolescents are included within broader population groups, with no explicit focus on their mental health challenges or service needs.

These findings suggest a policy vacuum that may contribute to limited political attention, fragmented programming, and inadequate investment in adolescent mental health services at both national and County levels.

##### Actor power—exclusion of adolescents and caregivers in mental health policy decisions

Participants highlighted critical imbalances in the exercise of actor power during adolescent mental health policy processes. Specifically, they emphasized that adolescents and their caregivers are systematically excluded from spaces where decisions are made, limiting their ability to influence priorities, policies, and programming. This exclusion reflects a concentration of decision-making authority among adult professionals and policy actors, with adolescents and caregivers relegated to passive roles despite being central to the issue.

This absence of voice and representation illustrates a form of structural powerlessness, where adolescents lack both access to policy forums and mechanisms to assert their perspectives. One youth mental health advocate captured this dynamic: “*Whenever people are reporting on your behalf it is never said with the same urgency, or it does not get as much attention as when the people themselves speak” (KII with youth mental health advocate—CM).*

Here, the symbolic power of voice—the ability to frame and prioritize one’s own experiences is denied, resulting in decisions that may not resonate with or reflect adolescents lived realities. The reliance on adult proxies, even well-meaning ones, reproduces hierarchies that silence or dilute adolescent agency.

A policymaker similarly acknowledged the limitations of these top-down approaches: “*We make decisions for people without including them to really understand what is it that they could be going through… we think that we have a solution for them but really it is a solution for us… when you give this solution to them it does not work, then we end up at the drawing table again” (KII with mental health specialists and educators—NA).*

This quote points to a misalignment of power and knowledge, where the absence of experiential insight from adolescents leads to ineffective or irrelevant policy solutions. Furthermore, participants noted that caregivers also remain largely excluded from policy processes, despite their influential role in shaping adolescents’ mental health outcomes.

As one participant observed: *“A parent should not be left out in my view because at the end of the day this adolescent is their child for the next so many years… that is also a stakeholder that needs to be brought on board” (KII with mental health specialists and educators—NA).*

The exclusion of caregivers represents a missed opportunity to mobilize relational and supportive forms of power that could enable more effective and sustainable interventions. In both cases, adolescents and caregivers are not only denied access to decision-making spaces but also deprived of the opportunity to exercise relational, participatory, and discursive power—forms that are essential to shaping responsive mental health systems.

#### Political contexts

##### Government transition and policy change

The often-changing political environment within Kenya, with repeated changes in policies and regulations, interferes with the sustainability of the already established adolescent mental health programs. A participant observed, “*I think in the last Government, there was a problem with how the executive engaged with parliamentarians, which I thought was very unfortunate. In previous governments, especially before 2013—you would see more synergy, even when a good proposal came from a private member” (KII with national-level MH policy specialists- KA).*

The challenge is also experienced in the devolved County Governments in Kenya, as highlighted by another participant, “*If you go to a county you will find that every five years you will find new people in the health system, so any activity that you start to discuss about adolescent and mental health, when you go back after five years you find the new persons or whoever have been employed as the CEC or the way they are running their system, they have changed and there is no record” (KII with mental health specialists and educators-DA).*

#### Issue characteristics

##### Insufficient data to quantify the mental health burden

The participants observed that there is a lack of sufficient data to quantify and estimate the real burden of adolescent mental health. Responding to the issues of prioritization at the policy level, a policy specialist said, “*I think the first step towards prioritizing adolescent mental health would be to estimate more accurately the burden of mental disease in this population …. as for clinical data there is none that we can get, routine data” (KII with National-level MH Policy specialists-CW).*

This was echoed by another participant, “*when it comes to data on issues of adolescent mental health I can say specifically on Kenya, we may not have enough data” (KII with National-level MH Policy specialists- SN).*

For mitigation, a participant suggested the need to start with a baseline survey and establish the magnitude of the adolescent mental health problem for effective planning. “*For me I feel we need to do a survey to first establish the extent of the problem and probably now quantify it in terms of finances and demonstrate now the burden to the economy and now also be able now to quantify the investments” (KII with County (sub-national level) policy and MH program specialists-KO).*

##### Low level of funding for mental health programs

According to the participants, funding for mental health is significantly low in the Country. There is a need to advocate for more investment at the policy level. A participant observed, “*Financing is still very minimal for mental health. I think that is what I began by stating, and now we need to look for a formula to raise awareness and advocate more for additional resources, so advocacy basically” (KII with County (sub-national level) policy and MH program specialists-KO).*

Another participant concurred, “*I can see like you know that kind of intentions to really want to help, but seemingly it is not matched by the resources given to this health care system to be able to really handle the mental health of adolescent” (KII with youth mental health advocates-ST).*

### Implementation level issues

#### Actor power

##### Inadequate coordinated efforts for coalition building on the implementation of adolescent mental health programs

Limited coordination among the actors poses a significant challenge in advancing adolescent mental health. The actors would include non-governmental organizations, advocacy groups, government entities, and religious organizations. A youth mental health advocate responded to the challenge of having a unified stakeholders’ voice by saying*: “The lack of a strong umbrella body in mental health means many of us are working independently, without knowing what others are doing” (KII with youth mental health advocate).*

A mental health expert concurred*, “(stakeholders) are not aligned that is what I was just saying because everybody is doing their own thing, the churches are doing their own thing, the organizations out there are doing their own thing” (KII with mental health specialists and educators-DA).*

Participants identified mistrust among stakeholders as a significant barrier to coalition building within the mental health sector. They reported that attempts to unify efforts across different actors—such as NGOs, advocates, and government entities—often falter due to skepticism regarding the intentions, credibility, or leadership styles of those initiating the collaborations. This mistrust leads to fragmented initiatives and prevents the formation of a cohesive advocacy movement. As one youth mental health advocate explained, “*The general mental health space has been very hard to bring under one umbrella. There is a lot of mistrust, so people are looking at who is starting this program. If they don't like the person, they don't join. So, even those that are there are still one-man shows*” (KII with youth mental health advocate—CM).

#### Ideas

##### Positioning issue—inadequate priming of the adolescent mental health agenda

The absence of a strong positioning of the adolescent mental health agenda has made it harder to address the problem coherently. Hence, there are considerable knowledge gaps in the understanding of the field of mental health that prevent an articulation of ideas. A participant commented, “*there is the lack of knowledge on mental health.” CW. “I also don't think that there is enough literacy on mental health among adolescents” (KII with youth mental health advocates-GO).*

Another participant highlighted that stakeholders have not built a strong adolescent mental health agenda since it is often overshadowed by other competing issues, like youth sports advancement. During social events, adolescent mental health is not given a priority, and this hinders the advancement in terms of advocacy and investments. A youth mental health advocate highlighted*, “Adolescent agenda is sort of swallowed up in the youth agenda … you may find that there are organizations speaking to adolescents during sports, for example, … or it's during the world cup then that is more likely to be framed as a sports activity and then whatever you did on mental health is secondary” (KII with youth mental health advocates-CM).*

##### Inadequate mental health knowledge

Participants highlighted a widespread knowledge gap regarding adolescent mental health, which was evident not only among adolescents but also among caregivers and the broader community. This gap contributes to misconceptions, stigma, and inadequate responses to adolescents experiencing mental health challenges. A youth mental health advocate emphasized: “*I don't think that there is enough literacy on mental health among adolescents” (KII with youth mental health advocates-GO)*. Another participant explained how adolescents are often misunderstood and unfairly judged: “*Look at what is the perception of adolescent mental health… it is assumed to be behavioral issues, seeking attention” (KII with youth mental health advocates-CM)*. Similarly, a county-level respondent noted: “*There is the lack of knowledge on mental health” (KII with CW)*. Together, these perspectives reflect the need for targeted mental health literacy interventions across multiple societal levels.

#### Issue characteristics

##### Persistent stigma around mental ill-health

The community stigmatizes adolescents with mental health disorders, and some people even use derogatory labels that are demeaning. This is a hindrance to the implementation of adolescent mental health programs. A participant observed, “*You would find someone saying, ‘there is a child in that family that has a ‘kawodo’ … when they say ‘kawodo’” we understand, either because they will be pointing to their heads, or the way they roll their eyes …“In Swahili then it would be ‘mwendazimu,’ (insane) that is the common one, the slang would be ‘chizi,’ (insane) when I was younger it was ‘Kreki,’ (insane) yes” (KII with youth mental health advocates-CM).*

Another participant commented, ‘*the biggest challenge we have here is the stigma, stigma about mental health many people don't want to be associated with issues to do with mental health’ (KII with County (sub-national level) policy and MH program specialists-EB).*

##### Inadequate access to effective and affordable mental health services

Participants identified a lack of dedicated adolescent mental health infrastructure and uneven distribution of skilled personnel as a major challenge to equitable service access and affordability. One youth mental health advocate noted: “*Human resources that specifically support mental health are scarce in the country, and their distribution mainly favors urban areas” (KII with youth mental health advocates-CM).*

Another health care worker concurred, “*apart from maybe the psychiatric unit at Meru here which handles the psychiatric cases now like a rehabilitation center for the substance abuse victims, a place for doing counseling, we don't have those facilities at the moment, and they all need to be put in place” (KII with County (sub-national level) policy and MH program specialists-DO).*

## Discussion

The analysis revealed that adolescent mental health in Kenya remains politically under-prioritized due to a lack of a dedicated policy, exclusion of adolescent voices in decision-making, weak actor coordination, limited funding, persistent stigma, and insufficient data. These challenges were compounded by frequent changes in political leadership and minimal awareness or framing of adolescent mental health as a distinct issue.

Our findings highlight critical gaps in addressing adolescent mental health in Kenya, echoing challenges reported in other low- and middle-income countries (LMICs). Using the Shiffman and Smith model, this study underscores the multifaceted barriers as described using the domains of actor power, ideas, political contexts, and issue characteristics.

These findings align with global literature emphasizing the need for inclusive, coordinated efforts to prioritize adolescent mental health (Shiffman *et al.* 2016, Shiffman 2017).

The exclusion of adolescents from decision-making processes mirrors observations in prior studies from Sub-Saharan Africa, where youth participation remains tokenistic. For example, a study by Seekles *et al*. (2023) highlights that the absence of youth voices leads to interventions lacking cultural relevance and efficacy (Seekles *et al.* 2023). Furthermore, the exclusion of families, as highlighted in our study, undermines sustainable solutions since families often provide the primary social support for adolescents. Studies from India and Bangladesh corroborate this, showing that family-centered mental health interventions yield better outcomes in adolescent populations (Mehra *et al.* 2022, Bookman *et al.* 2024).

The lack of coalition-building and mistrust among stakeholders echoes findings from LMICs, where fragmented efforts dilute advocacy impacts. Studies such as Murphy *et al.* (2021), Seekles *et al.* (2023), Ionescu *et al.* (2024), Mubeen *et al.* (2024) have identified that fragmented approaches to mental health advocacy hinder the establishment of a unified mental health agenda. Our findings suggest the urgent need to foster collaboration among stakeholders through transparent and inclusive processes to reduce mistrust and fragmentation.

Positioning issues, including inadequate priming of adolescent mental health, resonate with global evidence that adolescent-specific mental health needs are often overshadowed by broader youth or adult mental health agendas (Kieling *et al.* 2011, Patton *et al.* 2016). Studies from Ethiopia and Zambia demonstrate that donor-driven agendas frequently overshadow locally identified mental health priorities. For instance, Alem *et al.* (2008) found that donor-led mental health initiatives in Ethiopia often failed to align with community needs. This concern is echoed in subsequent analyses (Alem *et al.* 2008, Hanlon *et al.* 2019, Social Work Institute 2024), which further emphasize how externally imposed priorities can marginalize local perspectives in mental health policy and service delivery.

In Kenya, however, donor influence did not emerge as a dominant factor affecting adolescent mental health prioritization. Instead, the limited visibility of adolescent mental health appears more deeply rooted in sociocultural contexts, where mental health is often perceived as a foreign or secondary concern. This perception—shared across many African societies, Kenya included—underscores the need to frame mental health interventions in culturally relevant narratives (Jenkins *et al*. 2010, Hart and Norris 2024).

Political transitions and policy instability, highlighted as barriers in our study, are consistent with findings from other LMICs. A study in Iran demonstrated that frequent changes in leadership disrupt the continuity of health programs, leading to resource inefficiencies (Ghiasipour *et al.* 2017). Similarly, devolution in Kenya has compounded these challenges, with county-level variations creating inconsistencies in mental health service delivery (Barasa *et al.* 2017). Strengthening governance structures and institutional memory is critical to overcoming these obstacles.

Issue characteristics such as stigma, poverty, and insufficient data remain pervasive challenges. Stigma remains a pervasive barrier, with derogatory language and negative perceptions deterring adolescents from seeking help. This stigma is prevalent globally, where societal attitudes towards mental health issues contribute to underreporting and a lack of support (Doyle *et al.* 2022). Our findings align with other studies, where derogatory language perpetuates negative attitudes and impedes access to care (Kip *et al.* 2022, Sheikhan *et al.* 2023). Addressing stigma requires a multi-pronged approach, including community education, anti-stigma campaigns, and youth-driven advocacy.

Poverty and competing survival priorities further deprioritize adolescent mental health, as observed in our study and supported by evidence from LMICs (Wani *et al.* 2024). For example, adolescents living in multidimensionally poor households in Colombia were found to have about 50% higher risk of mental health problems compared to non-poor peers (Díaz *et al.* 2022). In rural Burkina Faso, socioeconomic status strongly predicted adolescents’ perception of need and healthcare utilization (Krohn *et al.* 2023). A 2024 systematic review across Africa also showed that hunger doubled the odds of mental health distress (Tinsae *et al.* 2024). Moreover, in Uganda, a poverty-reduction intervention reduced adolescent depression through improved family relationships (Karimli *et al*. 2023). Tackling this requires integrating mental health services into broader health and social welfare programs to ensure accessibility and affordability.

Inadequate access to effective and affordable mental health services, coupled with insufficient data on the adolescent mental health burden, hampers the development of targeted interventions. Low funding further reflects the minimal prioritization of mental health, a trend observed in many low-resource settings where health budgets are constrained (Warraitch *et al.* 2024).

Finally, insufficient data on adolescent mental health burden hampers evidence-based planning and resource allocation (Juma *et al.* 2020). This gap is consistent with findings from LMICs where routine data collection on mental health is inadequate (Juma *et al.* 2020). For example, Rwanda's success in improving HIV outcomes through robust data systems offers lessons for building comprehensive adolescent mental health databases (Binagwaho *et al.* 2014, Nsanzimana *et al.* 2015).

### Implications

The findings have several policy and programmatic implications. First, enhancing adolescent participation in mental health policymaking is paramount. This can be achieved through youth-led platforms and integrating adolescent voices in national and county-level decision-making processes (Yamaguchi *et al.* 2023, Kangwana *et al*. 2024). Second, strengthening family involvement in mental health programs is essential. Family-centered interventions, tailored to the unique sociocultural context, should be prioritized (Healy *et al*. 2018). Third, fostering coalition-building among stakeholders requires establishing transparent frameworks to reduce mistrust and encourage collaboration (Smith *et al.* 2021). A unified, locally owned adolescent mental health agenda will be instrumental in mobilizing resources and political support (Iemmi 2022).

Positioning adolescent mental health as a national priority demands targeted advocacy that frames it as integral to societal wellbeing (UNICEF 2020). Contextualizing mental health narratives in culturally resonant terms will enhance local ownership and reduce perceptions of foreign influence (Powell *et al*. 2023). Addressing political instability requires long-term strategies that institutionalize adolescent mental health programs, ensuring continuity across government transitions (Jenkins *et al.* 2011, Eaton 2019).

Tackling stigma and poverty requires a dual approach. Community-based anti-stigma campaigns led by adolescents, combined with economic empowerment programs, can shift perceptions and improve mental health outcomes (Waqas *et al.* 2020). Developing and implementing affordable, accessible mental health services, especially in low-resource settings, will also address critical gaps (Wainberg *et al.* 2017). Finally, investing in robust data systems for adolescent mental health is crucial. Establishing baseline data and routine surveillance mechanisms will guide evidence-based policy and program development (Shinde *et al.* 2023).

### Limitations

While this study provides valuable insights into the barriers hindering adolescent mental health prioritization in Kenya, several limitations should be acknowledged. First, as a qualitative study, the findings are context-specific and may not be generalizable to other regions or countries. The study relied on participant perspectives, which—though rich in depth and detail—may be influenced by recall bias or social desirability bias.

This study faced challenges in participant recruitment, which may have implications for the diversity and generalizability of the findings. Efforts were made to recruit a broad and inclusive range of stakeholders—including policy actors at national and County levels, youth advocates, mental health specialists, and educators—using both email and in-person invitations. Of the 32 individuals approached, only 15 ultimately participated. One declined, another was unable to participate due to illness, and 17 were unavailable due to time constraints linked to professional commitments and challenges related to the COVID-19 pandemic. While follow-up communications and flexible scheduling were employed to encourage participation, certain key stakeholder groups, particularly adolescents themselves and some high-level decision-makers, remained inaccessible. These limitations may have introduced selection bias. Nonetheless, the study captured a diverse mix of perspectives across multiple policy levels. Future research could consider longer recruitment timelines, the use of virtual interviews, or additional incentives to enhance participation and stakeholder representation.

The study also does not quantify the impact of specific barriers, which limits the ability to assess their relative influence on adolescent mental health policy and programming. Furthermore, while comparisons with other LMICs help to contextualize the findings, structural and sociocultural differences may affect their applicability elsewhere.

Finally, the use of the Shiffman and Smith (2007) framework to guide interpretation provided a useful structure for organizing emergent themes. However, this framework was originally developed for global health initiatives and prioritization at international levels. Applying it to Kenya’s national and sub-national contexts required adaptation, and some elements—such as global governance structures—were less relevant. At the same time, locally specific dynamics, such as devolved political structures and county-level transitions, emerged as particularly influential but are not explicitly captured in the framework. This underscores the importance of complementing such frameworks with inductive analysis and being mindful of contextual nuances when applying global models to local settings.

## Conclusion

This study highlights the complex interplay of factors hindering adolescent mental health prioritization in Kenya. Comparisons with other LMICs underscore the universality of these challenges, emphasizing the need for localized, inclusive, and coordinated strategies. Addressing these gaps will require strong political will, youth-driven advocacy, and sustained investment in mental health services. By framing adolescent mental health as a national priority, Kenya can pave the way for a healthier, more equitable future for its youth.

## Supplementary Material

czaf057_Supplementary_Data

## Data Availability

The data underlying this article will be shared at a reasonable request by the corresponding author.

## Reflexivity statement

Our research team comprises of a diverse group of authors with varying academic backgrounds, career stages, and regional affiliations, fostering a multidisciplinary and inclusive approach to the study.

The team includes early-career, mid-career, and senior researchers from multiple institutions across Kenya, the Netherlands, the United States, Sweden, and South Africa. This mix of expertise from psychology, psychiatry, public health, biostatistics and policy analysis ensures a comprehensive understanding of adolescent mental health policy in Kenya.
