# Supplementary material for: The political economy of adolescent mental health in Kenya
Source: Health Policy Plan. 2025 Aug 29;40(10):1017–26. doi: 10.1093/heapol/czaf057 (PMC12605744; doi:10.1093/heapol/czaf057)
Supplement: czaf057_Supplementary_Data [file czaf057_supplementary_data.docx]

**Appendix I: Key Informant Interview Guide- policy makers**

**Questionnaire for MH policy makers-**

**Key Informant Interview Guide (KII-G).**

## Generating global political priority for adolescent mental health and wellbeing informed by analysis of existing mental health policies, practices and services for adolescents. (AMH).

## *(Director of mental health, Chief Medical social worker, Psychologist)*

**General mental health aspect

Aim**: To increase knowledge of the political economy of adolescent mental health globally and Kenyan context.
**Objectives**: To investigate experts’ perceptions of adolescent mental health and wellbeing globally and locally in terms of; a) the topic being a global political priority, b) the *problem definition* and *positioning*, c) *governance* and *coalition-building* among stakeholders, and how this influences priority-setting and collective action.

**Research questions:**

- To what extent do experts perceive adolescent mental health and wellbeing as a global political priority?

*To get started, we’d like to ask you a few background questions about your personal life.*

**Opening questions**

1. How old are you? (Age in years) _____________________
2. Gender of respondent: a. Female b. Male c. Other
3. Marital Status
   1. Married
   2. Single (never married)
   3. Widowed
   4. Divorced/separated
4. How many children do you have? _____________________
5. What is the highest level of education you completed?
   1. No School
   2. Primary; last year completed: _____________________
   3. Secondary; last year completed: _____________________
   4. Completed Secondary
   5. Technical School; what in? _____________________
   6. University
   7. Who paid your school fees? _____________________
6. How many Ksh. does your household earn per month? ______ _______________
7. Who earns money for your household?
   1. Self
   2. Spouse/Partner
   3. Both
   4. State Grant
   5. Other ________________
8. What is your type of employment? How do you get money?

a. Self b. Casual Labor c. Permanent with Pension d. Contract e. Unemployed

1. What is your type of residence? Where do you live?

a. Tenant b. Own house c. Stay with family with no rent contribution

1. Have you ever been diagnosed of mental illness? a. Yes b. No
2. Has any member of your family/relative been diagnosed of mental illness? a. Yes b. No

**Broad introductory questions - level and development of priority**

1. In your opinion, what can you say is the achievement of;
   1. Mental health and wellbeing in terms of global political priority?
   2. Adolescent mental health and wellbeing in Kenyan political priority?

*Probe (What evidence would you cite in each of the above to support your statements?)*

1. To what extent do you think has the **global** political priority for adolescent mental health and wellbeing changed over time?
   1. In the last decades?
   2. In the last 1-2 years?
2. What do you think influences/challenges the above answers you have given in relation to adolescent MH in Kenyan context?
   1. To what extent does the global political priorities influence local MH adoption for AMH?
   2. How best can the challenges cited above be addressed to enhance adolescent MH?
3. What advocacy strategies do you think can be used to lobby for **more global** and **local political** prioritization for adolescent MH?
   1. In your opinion what role does/can the Government play in **global** and **local advocacy** for adolescents’ MH?
4. Please tell me a little bit about how you got into mental health policy making field in Kenya.
   1. What can you say about mental health policy implementation in Kenya?
   2. To what extent, if any, do you think the current National mental health policy has in terms of achieving adolescent mental health in Kenya?
   3. What are some of the identifiable gaps in the current mental health policy in terms of addressing adolescent MH and wellbeing locally? How best can these (cited gaps) be addressed?
   4. To what extent does the current political priorities for adolescent MH contribute or/and influence the gaps cited in terms of mental health policy implementation and service deliveries in Kenya

**Additional questions**

1. From your perspective, which dimensions of the problem of adolescent mental health and wellbeing make it challenging to achieve collective action to address it?
2. What features of the problem facilitate collective action?
3. When it comes to data and research:
4. What is your view on the available data and research on adolescent mental health and wellbeing?
5. What do you think are the challenges in measuring adolescent mental health?
6. Who would you recommend I interview to learn more?
7. Would you have any additional comments?
8. What can you say about the current mental health policy is helpful in your organizational areas of interest/work in relation to;
   1. Adolescent MH
   2. Why? Please give details
   3. In the process of development, did the policy address mental health issues comprehensively? Give reasons for your answer/response.
9. What role does the NGO world/Partners play in addressing mental health issues?
   1. What is their role in policy implementation?
   2. How well is the mental health agenda represented in the health coordination body, what are the gaps and opportunities?
10. What are the structures in place for Mental health service delivery? In each level, what are the gaps/challenges and opportunities?
    1. How can service delivery be improved at each level?
    2. What are the new opportunities?
11. What can you say about mental health care financing at communities and health facilities?
    1. Where are we and what are the challenges?
    2. How can we address these challenges?
12. Does the ministry or/and County currently provide direct financial support in the implementation of the mental health policy in the country?
    1. If yes, kindly describe the key areas of support, duration and geographic scope (if applicable)
13. What is the role of Division of mental health in the mobilization of resources to support the implementation of the mental health policy in the country?
    1. Kindly describe the strategies undertaken to mobilize resources, describing successes and challenges.
    2. What strategies and mechanisms put in place to address the challenges?
14. What monitoring mechanisms are currently in place on mental health conditions/illnesses?
    1. Are these mechanisms comprehensively addressing the mental illness segregation? Give reasons for your answer.
    2. kindly describe the models implementation of the current policy, types of innovation or research undertaken on mental health.
15. **Do you have any questions for me, or comment, related to our discussion?**

**Appendix II: Key Informant Interview Guide- Adolescent Champions**

Semi structured questionnaire interview guide:

(Adolescent Champions)

**Aim**: To increase knowledge of the political economy of adolescent mental health globally

**Objectives**: To investigate experts’ perceptions of adolescent mental health and wellbeing globally in terms of a) the topic being a global political priority, b) the *problem definition* and *positioning*, c) *governance* and *coalition-building* among stakeholders, and how this influences priority-setting and collective action.

**Opening questions**

1. How old are you? (Age in years) _____________________
2. Gender of respondent: a. Female b. Male c. Other
3. Marital Status
   1. Married
   2. Single (never married)
   3. Widowed
   4. Divorced/separated
4. How many children do you have? _____________________
5. What is the highest level of education you completed?
   1. No School
   2. Primary; last year completed: _____________________
   3. Secondary; last year completed: _____________________
   4. Completed Secondary
   5. Technical School; what in? _____________________
   6. University
   7. Who paid your school fees? _____________________
6. How many Ksh. does your household earn per month? ______ _______________
7. Who earns money for your household?
   1. Self
   2. Spouse/Partner
   3. Both
   4. State Grant
   5. Other ________________
8. What is your type of employment? How do you get money?

a. Self b. Casual Labor c. Permanent with Pension d. Contract e. Unemployed

1. What is your type of residence? Where do you live?

a. Tenant b. Own house c. Stay with family with no rent contribution

1. Have you ever been diagnosed of mental illness? a. Yes b. No
2. Has any member of your family/relative been diagnosed of mental illness? a. Yes b. No

**Research questions:**

1. How do experts define and position the problem of adolescent mental health and wellbeing globally?

1. What is your view point on adolescent mental health prioritization in health care systems?
2. What are some specific needs for the adolescent mental health?
3. Who do you think is responsible for addressing adolescent mental health care needs?
4. Are there specific policies targeting adolescent mental health? If yes, how is the implementation process?
5. What is the level of involvement in adolescent mental health at individual, family, and community levels?
6. What is your experience on adolescent mental health promotion, prevention and treatment in the community?
7. In your viewpoint, how can these (promotion, prevention and treatment) be effectively coordinated in the health care system?
8. What is the relationship between mental health and other health condition (CD and NCDs)? And in your opinion, what should inform an appropriate approach in addressing adolescent mental health, CDs and NCDs in our health care systems?
9. What is your opinion on stakeholders’ commitment in adolescent mental health?
10. In your viewpoint, what is the level of investment in terms of mental health infrastructure?
11. What are the barriers in adolescent mental health? (Probe)
12. What are the solutions to the barriers you have mentioned on the question above? (Cite some examples if any and probe)
13. Any additional comment?

**Semi structured interview questionnaire guide:**

# **Appendix III: Key Informant Interview Guide-Governance and coalition-building among stakeholders**

**Aim**: To increase knowledge of the political economy of adolescent mental health globally

**Objectives**: To investigate experts’ perceptions of adolescent mental health and wellbeing globally in terms of a) the topic being a global political priority, b) the *problem definition* and *positioning*, c) *governance* and *coalition-building* among stakeholders, and how this influences priority-setting and collective action.

**Opening questions**

1. How old are you? (Age in years) _____________________
2. Gender of respondent: a. Female b. Male c. Other
3. Marital Status
   1. Married
   2. Single (never married)
   3. Widowed
   4. Divorced/separated
4. How many children do you have? _____________________
5. What is the highest level of education you completed?
   1. No School
   2. Primary; last year completed: _____________________
   3. Secondary; last year completed: _____________________
   4. Completed Secondary
   5. Technical School; what in? _____________________
   6. University
   7. Who paid your school fees? _____________________
6. How many Ksh. does your household earn per month? ______ _______________
7. Who earns money for your household?
   1. Self
   2. Spouse/Partner
   3. Both
   4. State Grant
   5. Other ________________
8. What is your type of employment? How do you get money?

a. Self b. Casual Labor c. Permanent with Pension d. Contract e. Unemployed

1. What is your type of residence? Where do you live?

a. Tenant b. Own house c. Stay with family with no rent contribution

1. Have you ever been diagnosed of mental illness? a. Yes b. No
2. Has any member of your family/relative been diagnosed of mental illness? a. Yes b. No

**Research questions:**

1. How do governance and coalition-building among stakeholder’s influence priority-setting and collective action for adolescent mental health and wellbeing globally, according to experts?

**Stakeholders worried about adolescent mental health and wellbeing**

**I. Problem definition**

1. I use the term “adolescent mental health and wellbeing” during this interview, although I realize that you may use different words to describe this area.
2. Which terminology do you use?
3. How do you define the problem of adolescent mental health and wellbeing?
4. Who do you perceive as the main stakeholders engaged in adolescent mental health and wellbeing globally, i.e., individuals or organizations with an interest in the topic?
5. To what extent do you think stakeholders’ views are aligned when they speak about the problems and solutions regarding adolescent mental health and wellbeing, and its solutions? Where do they differ? Overlap?

**II. Governance and coalition-building**

1. From your perspective, how does the global community of stakeholders in adolescent mental health and wellbeing look like?
2. Which stakeholders make up this community?
3. To what extent do you think stakeholders at the grassroots (e.g., implementers) level are involved?
4. To what extent are adolescents involved?
5. In how far do you think the global community of stakeholders constitutes a unified community?
6. Have you encountered any stakeholders that oppose the prioritization of adolescent mental health and wellbeing?
7. Which stakeholders? Why?
8. How well has this opposition been handled?
9. In terms of leadership for adolescent mental health and wellbeing globally, is there any individual who you consider a leader for adolescent mental health and wellbeing globally?
10. How has this leader been able to convene stakeholders to strengthen adolescent mental health and wellbeing worldwide?
11. In your opinion, is there any organization that you would consider a leader in strengthening adolescent mental health and wellbeing globally?
12. How has this organization been able to convene stakeholders to strengthen adolescent mental health and wellbeing worldwide? What makes them an effective leader?
13. Thinking outside the field of adolescent mental health and wellbeing and the health sector, in how far have stakeholders forged alliances with those outside the field?

**III. Framing/positioning**

1. When it comes to positioning adolescent mental health and wellbeing, how have stakeholders framed the problem for decision-makers (e.g., in governments, international and funding organizations)?
2. How well have stakeholders shown how serious the problem is?
3. How convincing are the solutions that stakeholders have proposed?
4. How effectively have stakeholders communicated solutions to attract political support?
5. From your point of view, how effective have stakeholders been in increasing the priority of adolescent mental health and wellbeing among decision-makers?
6. Do you have examples of stakeholders that have shifted priorities towards adolescent mental health and wellbeing?

**Semi structured interview questionnaire guide:**

# **Appendix IV Key Informant Interview Guide: Key Politicians**

**Aim**: To increase knowledge of the political economy of adolescent mental health globally

**Objectives**: To investigate experts’ perceptions of adolescent mental health and wellbeing globally in terms of a) the topic being a global political priority, b) the *problem definition* and *positioning*, c) *governance* and *coalition-building* among stakeholders, and how this influences priority-setting and collective action.

*To get started, we’d like to ask you a few background questions about your personal life.*

**Opening questions**

1. How old are you? (Age in years) _____________________
2. Gender of respondent: a. Female b. Male c. Other
3. Marital Status
   1. Married
   2. Single (never married)
   3. Widowed
   4. Divorced/separated
4. How many children do you have? _____________________
5. What is the highest level of education you completed?
   1. No School
   2. Primary; last year completed: _____________________
   3. Secondary; last year completed: _____________________
   4. Completed Secondary
   5. Technical School; what in? _____________________
   6. University
   7. Who paid your school fees? _____________________
6. How many Ksh. does your household earn per month? ______ _______________
7. Who earns money for your household?
   1. Self
   2. Spouse/Partner
   3. Both
   4. State Grant
   5. Other ________________
8. What is your type of employment? How do you get money?

a. Self b. Casual Labor c. Permanent with Pension d. Contract e. Unemployed

1. What is your type of residence? Where do you live?

a. Tenant b. Own house c. Stay with family with no rent contribution

1. Have you ever been diagnosed of mental illness? a. Yes b. No
2. Has any member of your family/relative been diagnosed of mental illness? a. Yes b. No

**Qualitative questions**

*Broad introductory questions - level and development of priority*

1. In your opinion, how far is mental health and wellbeing a global political priority?
2. Specifically, how far is adolescent mental health and wellbeing a global political priority? Probe - What evidence would you cite to support your assertion?
3. How do you think has the global political priority for mental health and wellbeing changed over time?
   1. In the last decades?
   2. In the last 1-2 years?
4. What about your view point on mental health political prioritization in the country? (What has changed in the last decade, the last 1-2 years?)
5. What are the specific needs for the adolescent mental health and how should they be addressed?
6. From your perspective, how does the global community of stakeholders in adolescent mental health and wellbeing look like?
   1. Probe - Who do you perceive as the main stakeholders engaged in adolescent mental health and wellbeing globally, i.e., individuals or organizations with an interest in the topic?
7. Who do you think is responsible for addressing adolescent mental health care needs in the country?
8. What is your opinion on stakeholders’ commitment in adolescent mental health?
9. To what extent do you think stakeholders at the grassroots (e.g., implementers) level are involved? What is the level of involvement of the adolescents, family and community?
10. To what extent do you think stakeholders’ views are aligned when they speak about the problems and solutions regarding adolescent mental health and wellbeing, and its solutions?

Probe: Where do they differ? Where do they overlap?

Probe - Have you encountered any stakeholders that oppose the prioritization of adolescent mental health and wellbeing? Which stakeholders? Why? How well has this opposition been handled?

1. When it comes to positioning adolescent mental health and wellbeing, how have stakeholders framed the problem for decision-makers (e.g., in governments, international and funding organizations)?

Probe - How well have stakeholders shown how serious the problem is?

Probe - How convincing are the solutions that stakeholders have proposed?

Probe - How effectively have stakeholders communicated solutions to attract political support?

1. What are the barriers in adolescent mental health?
2. What are the solutions to the barriers you have mentioned on the question above? (Cite some examples if any)
3. When it comes to data and research:

What is your view on the available data and research on adolescent mental health and wellbeing?

What do you think are the challenges in measuring adolescent mental health?

1. Which are the specific policies targeting adolescent mental health in the Country? How is the implementation process of these policies?
2. Any additional comments?
